# *Brca2* and *Trp53* Deficiency Cooperate in the Progression of Mouse Prostate Tumourigenesis

**DOI:** 10.1371/journal.pgen.1000995

**Published:** 2010-06-24

**Authors:** Jeffrey C. Francis, Afshan McCarthy, Martin K. Thomsen, Alan Ashworth, Amanda Swain

**Affiliations:** 1 Section of Gene Function and Regulation, Institute of Cancer Research, London, United Kingdom; 2 Breakthrough Breast Cancer Research Centre, Institute of Cancer Research, London, United Kingdom; Stanford University School of Medicine, United States of America

## Abstract

Epidemiological studies have shown that one of the strongest risk factors for prostate cancer is a family history of the disease, suggesting that inherited factors play a major role in prostate cancer susceptibility. Germline mutations in *BRCA2* predispose to breast and ovarian cancer with its predominant tumour suppressor function thought to be the repair of DNA double-strand breaks. *BRCA2* has also been implicated in prostate cancer etiology, but it is unclear the impact that mutations in this gene have on prostate tumourigenesis. Here we have undertaken a genetic analysis in the mouse to determine the role of *Brca2* in the adult prostate. We show that deletion of *Brca2* specifically in prostate epithelia results in focal hyperplasia and low-grade prostate intraepithelial neoplasia (PIN) in animals over 12 months of age. Simultaneous deletion of *Brca2* and the tumour suppressor *Trp53* in prostate epithelia gave rise to focal hyperplasia and atypical cells at 6 months, leading to high-grade PIN in animals from 12 months. Epithelial cells in these lesions show an increase in DNA damage and have higher levels of proliferation, but also elevated apoptosis. Castration of *Brca2;Trp53* mutant animals led to regression of PIN lesions, but atypical cells persisted that continued to proliferate and express nuclear androgen receptor. This study provides evidence that *Brca2* can act as a tumour suppressor in the prostate, and the model we describe should prove useful in the development of new therapeutic approaches.

## Author Summary

In Western countries, prostate cancer is the most common male cancer and the second biggest cause of cancer-related deaths in men. Men with a familial history of either breast or ovarian cancer have an elevated predisposition to prostate cancer, suggesting there is a genetic element to this disease. Indeed, the inheritance of a mutated form of the breast cancer susceptibility gene *BRCA2* has been linked to the development of prostate cancer, although the precise role that BRCA2 dysfunction plays in the development of prostate cancer is unclear. To address this, we have generated an animal model in which the mouse *Brca2* gene is specifically deleted in the adult prostate. These mice develop precancerous prostate lesions, which progress in severity and incidence with the loss-of-function of an additional tumour suppressor, *Trp53*. Importantly, blocking male steroidal hormone production by castration leads to partial regression of the prostate lesions, however cells continue to proliferate after androgen withdrawal. This suggests human *BRCA2* mutant prostate tumours, like the majority of prostate cancers, will respond to hormone therapy, but will relapse, as frequently occurs in this disease. In summary, our model suggests that *BRCA2* acts as a tumour suppressor in the prostate and provides a pre-invasive model to test novel therapeutics.

## Introduction

Prostate cancer is the most common cancer in men in developed countries, with a rising incidence of the disease. However, the etiology of this malignancy is still unclear. Prostate cancer progresses through a pathologically defined series of steps involving increasing grades of PIN, invasive adenocarcinoma and metastatic cancer [1]. Androgens are crucial for normal prostate function, and act as pro-survival and proliferation factors in cancer cells. As such, prostate cancer is sensitive to androgen levels and androgen depletion therapy via chemical or surgical castration is an initial step in treatment, typically resulting in tumour regression. However, the cancer normally re-grows and develops as a castration-independent tumour.

Epidemiological studies have shown that one of the strongest risk factors for prostate cancer is a family history of the disease, suggesting that inherited factors play a major role in prostate cancer susceptibility [2], [3]. Approximately 10% of prostate cancers are thought to be hereditary, and this number increases with early on-set disease. In spite of this, little is known about the mechanisms of tumourigenesis of inherited prostate cancer. Prostate cancer frequently clusters in families that have breast cancer, indicating a genetic link between these two diseases [4]–[6]. Germline mutations in *BRCA2* predispose to both breast and ovarian cancer making it a good candidate gene for prostate cancer etiology. There is an increased risk of prostate cancer in individuals carrying a mutation in *BRCA2*, particularly early-onset disease [7]–[10]. The Breast Cancer Linkage Consortium found a significant relative risk of 4.65 for prostate cancer in male carriers of a deleterious *BRCA2* mutation that rose to 7.33 in men under 65 years of age [7]. Consistent with this, analysis of men with early-onset disease indicates that *BRCA2* carriers account for between 0.8–2% of prostate cancer cases, compared with the prevalence of 0.1% *BRCA2* mutations in the general population [11], [12]. In addition, *BRCA2* mutation carriers have been associated with aggressive prostate cancer [13]–[16].

BRCA2 is thought to act as a tumour suppressor, with tumours arising from *BRCA2* mutations frequently demonstrating loss-of-heterozygosity with loss of the wild-type allele. BRCA2 plays an important role in the repair of DNA double-strand breaks (DSB) through homologous recombination (HR) [17]. When there is a second identical DNA copy (i.e. the sister chromatid after replication) HR is the primary method of repair and is a relatively error-free DNA repair pathway. After DNA damage, BRCA2 directly interacts with the recombinase RAD51, a process that is essential for HR-mediated repair of DSBs [18]. When HR is defective or no sister chromatid is available the error-prone methods of single-strand annealing and non-homologous end joining are used for DNA repair [19]. *BRCA2*-deficient cells form chromosomal aberrations spontaneously in culture and are more sensitive to certain DNA damaging agents [19]–[21]. Hence, loss of *BRCA2* is thought to principally lead to tumour progression by the failure to repair DNA by HR, leading to genomic instability.

Mouse models have shown a direct *in vivo* tumour suppressor role for *Brca2* in the mammary gland and have demonstrated a synergistic tumour suppressor activity with *Trp53*. However, *Brca2* heterozygous animals do not show a predisposition to tumour formation and *Brca2* null mice result in embryonic lethality [18], [22], [23]. To circumvent this prenatal lethality, the *Cre-LoxP* system has been used to conditionally delete *Brca2* in a tissue-specific manner. Deletion of *Brca2* from the mouse mammary epithelium either fails to produce mammary-gland tumours or results in mammary-gland tumour formation with long latency (1.4–1.6 years) [24]–[26]. Tumour latency was reduced in *Brca2* mutant mice that were *Trp53* heterozygous [24]. In addition, mice with conditional inactivation of *Brca2* and *Trp53* developed mammary tumours with high penetrance at 6 months [25].

To understand the role of *Brca2* in prostate cancer we have used a prostate–specific *Cre* line and a conditional *Brca2* allele to delete *Brca2* in adult mouse prostate epithelia. We show that loss of *Brca2* in the prostate results in focal hyperplasia and low-grade (LG) PIN. Mice with conditional deletion of *Brca2* and *Trp53* have a high incidence of high-grade (HG) PIN, which contain cells with elevated DNA damage. PIN lesions in *Brca2;Trp53* homozygous mutant prostates persist and continue to proliferate after androgen depletion. This work confirms the role of *Brca2* as a tumour suppressor in the prostate and provides a model to test potential therapeutics in *Brca2*-deficient prostate neoplasia.

## Results

### Deletion of *Brca2* from prostate epithelia results in hyperplasia and low-grade PIN

To investigate the role of *Brca2* in the prostate we deleted *Brca2* from the adult mouse prostate epithelia. To achieve this we mated mice carrying a *Brca2* allele that has exon 11 flanked by loxP sites (*Brca2^F/F^*) to transgenic mice carrying *Cre* recombinase under the control of a prostate-specific composite rat probasin promoter, *PBCre4* [25], [27]. This *Cre* line has been used successfully to delete tumour suppressor genes and activate oncogenes to drive prostate neoplasia and tumour progression [28]–[30]. Deletion of this *Brca2* conditional allele results in the loss of a Rad51-interacting domain, and consequently, homozygous germline deletion leads to embryonic lethality [25].

**Figure 1:**
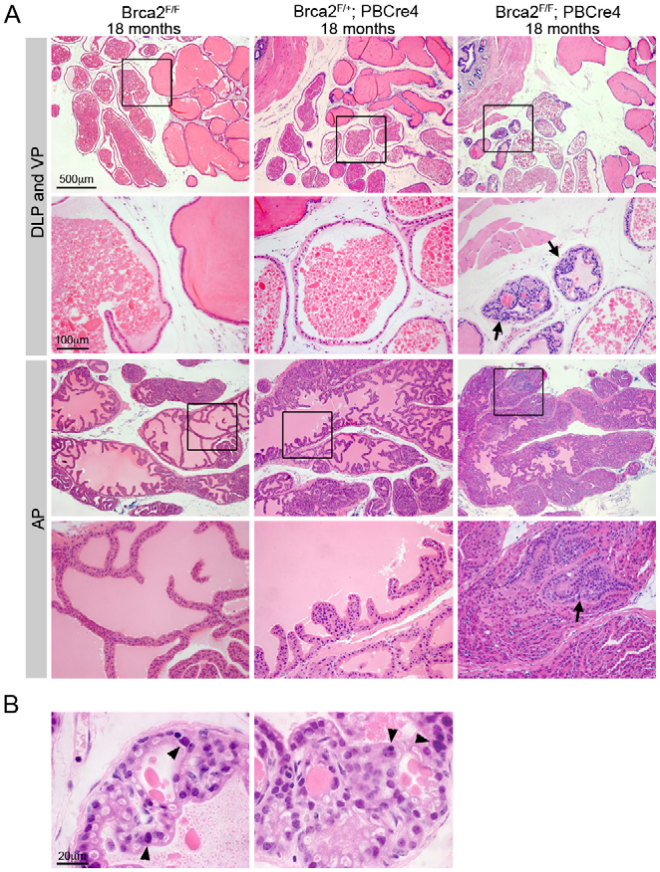
Deletion of *Brca2* from prostate epithelia results in hyperplasia and LG PIN. (A) Haematoxylin and eosin stained sections of dorsolateral prostate (DLP), ventral prostate (VP) and anterior prostate (AP) of control and *Brca2* mutants. Control (*Brca2^F/F^*) and *Brca2* heterozygous (*Brca2^F/+^;PBCre4*) prostates do not have PIN. Prostate-specific homozygous deletion of *Brca2* (*Brca2^F/F^;PBCre4*) results in focal hyperplasia and LG PIN (indicated with arrows). Black box shows the area shown in higher magnification in the panel below. (B) Detail of LG PIN in the DLP of *Brca2* mutant prostates. Left panel shows a lumen with tufting pattern and right panel shows cribiform pattern. Arrowheads indicate atypical cells.

Cohorts of male control (*Brca2^F/F^*), *Brca2* heterozygous (*Brca2^F/+^;PBCre4*) and *Brca2* mutant (*Brca2^F/F^;PBCre4*) animals were generated and analysed for tumour progression at 6 months, 10–14 months and 15–20 months of age. None of the *Brca2^F/+^;PBCre4* prostates had any observable morphological differences compared to control prostates at any time point analysed (Figure 1 and Table 1). Focal hyperplasia that contained atypical cells was first observed in *Brca2^F/F^;PBCre4* prostates at 10–14 months and was also present in these animals at 15–20 months (Table 1). In addition, at 15–20 months a significant number of *Brca2* homozygous mutant prostates had focal LG PIN in their lumen compared to control animals (6/17 compared to 0/28; Z-test p = 0.0001) (Figure 1A and Table 1). LG PIN lesions formed characteristic tufting and cribiform patterns (Figure 1B). These focal lesions contained multiple atypical cells that had prominent nucleoli and hyperchromasia. Hyperplasia and LG PIN lesions were present in all four prostatic lobes of *Brca2* mutants.

**Table 1:** The number of control, *Brca2* mutant and *Brca2*;*Trp53* mutant prostates analysed and their phenotype.

Genotype	Age	Total	Hyp	LG PIN	HG PIN
*Control*	6 months	4	0	0	0
	10–14 months	11	0	0	0
	15–20 months	28	2	0	0
*Brca2^F/+^;PBCre4*	12–20 months	7	1	0	0
*Brca2^F/F^;PBCre4*	6 months	2	0	0	0
	10–14 months	4	2	0	0
	15–20 months	17	10	6	0
*Brca2^F/F^;Trp53^F/+^;PBCre4*	6 months	2	0	0	0
	10–14 months	10	7	2	0
	15–20 months	15	11	8	6
*Brca2^F/F^;Trp53^F/F^;PBCre4*	6 months	3	2	0	0
	10–14 months	15	12	9	7
	15–19 months	32	30	29	27

*Brca2* homozygous mutant prostates (*Brca2^F/F^;PBCre4*) have hyperplasia (Hyp) at 10–14 months and LG PIN at 15–20 months. *Brca2* homozygous *Trp53* heterozygous mutant prostates (*Brca2^F/F^;Trp53^F/+^;PBCre4*) have hyperplasia and LG PIN at 10–14 months, and by 15–20 months have HG PIN. *Brca2*;*Trp53* double homozygous mutant (*Brca2^F/F^;Trp53^F/F^;PBCre4*) prostates have hyperplasia at 6 months and LG PIN at 10–14 months, and by 15–19 months have a high incidence of LG PIN and HG PIN.

### Combined deletion of *Brca2* and *Trp53* leads to frequent high-grade PIN

The tumour suppressor *TP53* is frequently mutated in *BRCA2* cancers and studies in the mouse have shown a genetic interaction between *Brca2* and *Trp53* [24], [31], [32]. To test if *Brca2* and *Trp53* cooperate in the prostate we deleted both of these genes in the prostate epithelia using the *PBCre4* transgene. Cohorts of male control (*Brca2^F/F^;Trp53^F/F^*), *Brca2* homozygous and *Trp53* heterozygous (*Brca2^F/F^;Trp53^F/+^;PBCre4*) and *Brca2* and *Trp53* double homozygous (*Brca2^F/F^;Trp53^F/F^;PBCre4*) animals were generated and analysed for tumour progression.

**Figure 2:**
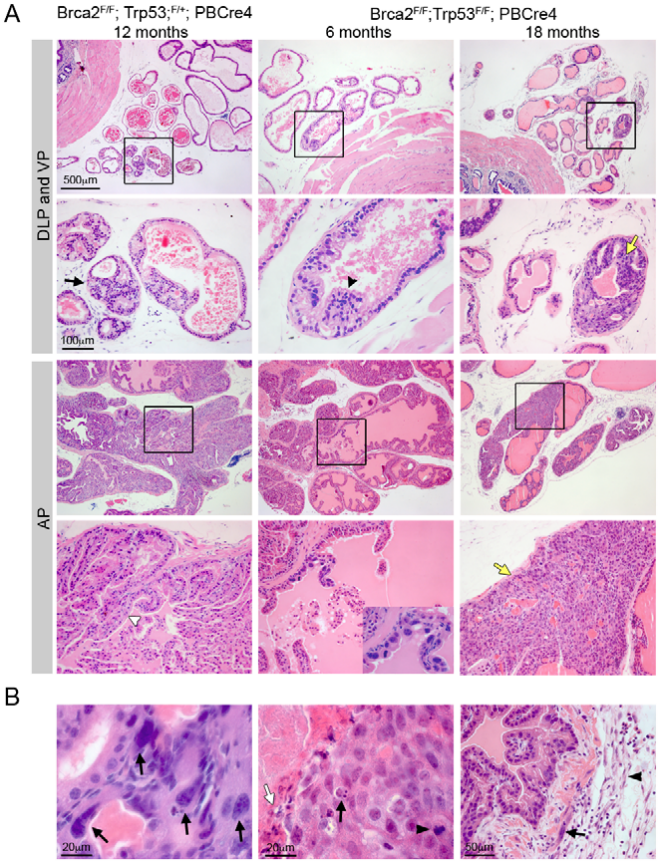
Combined deletion of *Brca2* and *Trp53* leads to HG PIN. (A) Haematoxylin and eosin stained sections of dorsolateral prostate (DLP), ventral prostate (VP) and anterior prostate (AP) of *Brca2;Trp53* mutants. Homozygous deletion of *Brca2* and heterozygous loss of *Trp53* (*Brca2^F/F^;Trp53^F/+^;PBCre4*) in prostate epithelia results in hyperplasia (indicated with white arrowhead) and PIN (indicated with black arrow). Prostate-specific homozygous deletion of *Brca2* and *Trp53* (*Brca2^F/F^;Trp53^F/F^;PBCre4*) results in hyperplasia (indicated with black arrowhead) and atypical cells at 6 months of age. Close-up in bottom panel shows cluster of atypical cells. *Brca2;Trp53* homozygous mutants have focal HG PIN (indicated with yellow arrows). Black box shows the area that is shown in higher magnification in the panel below. (B) Detail of HG PIN in the AP of *Brca2;Trp53* mutants. Left panel shows atypical cells with arrows indicating severe pleomorphic nuclei. Middle panel shows a mitotic figure (black arrowhead), apoptotic body (black arrow) and an area of necrosis (white arrow). Right panel shows epithelial cells protruding into broken-up atypical smooth muscle cells (black arrow) and desmoplastic stroma (black arrowhead).

In addition to hyperplasia and LG PIN observed in *Brca2* mutants, deletion of *Brca2* and *Trp53* resulted in the formation of HG PIN lesions. At 10–14 months, *Brca2^F/F^;Trp53^F/+^;PBCre4* animals had focal LG PIN and hyperplasia (Figure 2A and Table 1). By 15–20 months, LG PIN was still present and a significant number of animals had focal HG PIN compared to control animals (6/15 compared to 0/28; Z-test p = 0.0017). However, the frequency of HG PIN was significantly higher in *Brca2^F/F^;Trp53^F/F^;PBCre4* animals compared to *Brca2^F/F^;Trp53^F/+^;PBCre4* animals at this age (27/32 compared to 6/15; Z-test p = 0.0058) (Figure 2A and Table 1). In these animals, focal areas of hyperplasia consisting of atypical cells were present as early as 6 months. At 10–14 months, LG PIN was present and a significant number of *Brca2^F/F^;Trp53^F/F^;PBCre4* animals had HG PIN compared to control animals (7/15 compared to 0/11; Z-test p = 0.0276). Hyperplasia and LG PIN lesions were similar to those found in *Brca2^F/F^;PBCre4* mutants. Frequently multiple ducts of each lobe had HG PIN, which were present in proximal and distal regions of the prostate and consisted of many atypical cells filling the lumen. Atypical cells were unorganised with poor orientation, severe nuclear pleomorphism and abnormal nuclear to cytoplasm ratios (Figure 2B). Mitotic figures, apoptotic bodies and areas of necrosis were also present within HG PIN lesions (Figure 2B). In some cases, epithelial cells of the lumen protrude into the adjacent stroma and the smooth muscle surrounding the ducts was no longer continuous but was broken up (Figure 2B). These areas contained atypical smooth muscle cells and desmoplasia in the surrounding stroma. HG PIN lesions were predominantly seen in the anterior prostate (AP) and dorsal prostate (DP) of *Brca2;Trp53* homozygous mutant animals, with a small number observed in lateral and ventral lobes. Deletion of LoxP flanked *Brca2* and *Trp53* alleles by the *PBCre4* transgene in the prostate was confirmed by PCR analysis on micro dissected tissue (Figure S1).

### *Brca2;Trp53* HG PIN lesions display increased DNA damage and apoptosis

**Figure 3:**
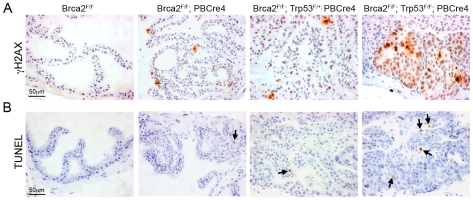
*Brca2;Trp53* mutants have increased DNA damage and apoptosis. (A) Immunohistochemistry for γH2AX shows several cells have DNA damage in LG PIN areas in *Brca2^F/F^;PBCre4* and *Brca2^F/F^;Trp53^F/+^;PBCre4* mutant prostates, whereas control (*Brca2^F/F^*) prostates have undetectable levels of DNA damage. *Brca2^F/F^;Trp53^F/F^;PBCre4* mutants have multi-focal areas with a large number of cells with DNA damage in regions of HG PIN. (B) TUNEL assay on sections of prostates demonstrates *Brca2^F/F^;PBCre4* and *Brca2^F/F^;Trp53^F/+^;PBCre4* mutant prostates have an increased level of apoptosis, compared to control (*Brca2^F/F^*) prostates. The level of apoptotic cells increases further in HG PIN lesions of *Brca2^F/F^;Trp53^F/F^;PBCre4* mutants. Arrows indicate apoptotic cells. The anterior prostates of 16-month-old animals are shown.

As the predominant tumour suppressor function of BRCA2 is thought to be the repair of DNA DSBs, we assessed the level of spontaneous DNA damage in *Brca2* and *Brca2;Trp53* mutant prostates. An early response to DNA damage is the phosphorylation of histone H2AX (γH2AX) [33]. Areas of hyperplasia and PIN in *Brca2* mutant and *Brca2;Trp53* mutant prostates contained cells that were positive for γH2AX, which were not present in control prostates (Figure 3A). While *Brca2^F/F^;PBCre4* and *Brca2^F/F^;Trp53^F/+^;PBCre4* LG PIN lesions had individual or small groups of γH2AX positive cells, *Brca2;Trp53* homozygous mutant prostates had large focal areas with many positive cells. These areas of γH2AX correlated with focal HG PIN lesions and were predominantly present in the AP and DP.

Deletion of *Brca2* frequently results in increased levels of cellular apoptosis, presumably as a result of increased constitutive DNA damage. To determine the level of apoptosis in prostate epithelia after deletion of *Brca2* and *Trp53* we used the TUNEL assay, which has been used to identify apoptosis in *Brca2* null neural tissue [32]. *Brca2* mutant prostates showed a 3 fold increase in TUNEL positive cells in areas of hyperplasia and LG PIN, compared to control prostates that had few apoptotic cells (0.3% TUNEL positive cells vs 0.1% in control) (Figure 3B). A 4 fold increase in apoptosis was observed in *Brca2^F/F^;Trp53^F/+^;PBCre4* PIN foci (0.4% TUNEL positive cells vs 0.1% in control). Notably, there was a 20 fold increase in apoptotic cells in areas of HG PIN in *Brca2;Trp53* homozygous mutant prostates (2% TUNEL positive cells vs 0.1% in control) (Figure 3B). An anti-Caspase-3 antibody and histological analysis confirmed that TUNEL positive cells were apoptotic and not the result of labelling damaged DNA (data not shown).

### *Brca2;Trp53* mutant prostates have hallmarks of cancer

**Figure 4:**
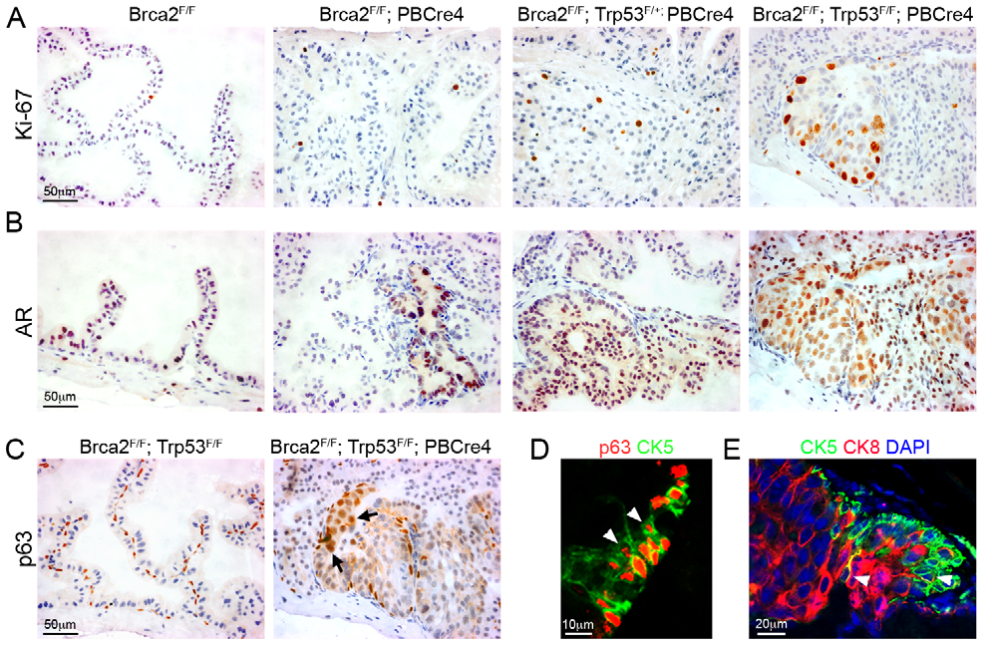
*Brca2;Trp53* mutant prostates have hallmarks of cancer. (A) Ki-67 immunohistochemistry shows increased proliferation in areas of LG PIN in *Brca2^F/F^;PBCre4* and *Brca2^F/F^;Trp53^F/+^;PBCre4* mutant prostates, a low number of proliferating cells are present in control (*Brca2^F/F^*) prostates. *Brca2^F/F^;Trp53^F/F^;PBCre4* mutants have a large number of proliferating cells in areas of HG PIN. (B) AR immunohistochemistry demonstrating increased levels of expression throughout the nucleus and cytoplasm in areas of LG PIN in *Brca2^F/F^;PBCre4* and *Brca2^F/F^;Trp53^F/+^;PBCre4* mutant prostates, compared to control prostates (*Brca2^F/F^*). *Brca2^F/F^;Trp53^F/F^;PBCre4* mutants have increased AR expression predominantly in the nucleus of cells in areas of HG PIN. (C) p63 immunohistochemistry shows an increase in p63-expressing cells in HG PIN lesions in *Brca2^F/F^;Trp53^F/F^;PBCre4* mutant prostates and normal expression in the basal cells of control (*Brca2^F/F^;Trp53^F/F^*) prostates. Arrows indicate a cluster of abnormal p63-expressing cells that are rounder and nearer the lumen. (D) Left panel shows p63 (red) and CK5 (green) fluorescent immunohistochemistry analysis with labelled cells protruding into the lumen (white arrowheads) in a region of PIN in *Brca2^F/F^;Trp53^F/F^;PBCre4* mutants. Right panel shows CK5 (green, basal cells) and CK8 (red, luminal cells) fluorescent immunohistochemistry with PIN lesions in *Brca2^F/F^;Trp53^F/F^;PBCre4* mutants displaying an increase in luminal cells next to clusters of basal cells. White arrowhead marks CK5 and CK8 double-labelled cells. DAPI nuclear stain is blue. The anterior prostates of 16-month-old animals are shown.

Ki-67 is a marker of proliferating cells and a prognostic indicator in prostate cancer [34]. Analysis of Ki-67 showed a low number of proliferating cells in control prostates that increased 6 fold in areas of hyperplasia and LG PIN in *Brca2^F/F^;PBCre4* mutant prostates (2.2% Ki-67 positive cells vs 0.4% in control) (Figure 4A). Levels of proliferation were 9 fold higher in *Brca2^F/F^;Trp53^F/+^;PBCre4 PIN* lesions (3.5% Ki-67 positive cells vs 0.4% in control), and dramatically increased by 30 fold in *Brca2^F/F^;Trp53^F/F^;PBCre4* HG PIN lesions (12% Ki-67 positive cells vs 0.4% in control) (Figure 4A).

Levels of the androgen receptor (AR) expressed in the luminal epithelium usually increase in the nucleus during human prostate carcinoma progression [35]. *Brca2^F/F^;PBCre4* and *Brca2^F/F^;Trp53^F/+^;PBCre4* mutant prostates had increased AR expression in the cytoplasm and nucleus of epithelial cells that correlated with regions of LG PIN (Figure 4B). The level of AR increased significantly more in *Brca2^F/F^;Trp53^F/F^;PBCre4* HG PIN lesions where it was found predominantly in the nucleus of luminal epithelial cells (Figure 4B).

The human and mouse prostate comprise of basal, luminal and rare neuroendocrine epithelial cells. In addition, intermediate or transit amplifying (TA) cells are observed in human prostates [36]. *Brca2;Trp53* homozygous mutant HG PIN lesions frequently contained large groups of p63-expressing cells, a marker of basal cells (Figure 4C). Instead of their normal flat shape and position basal to the luminal cells, some p63-expressing cells were rounder and in a position near the lumen of the prostate. Sections fluorescently double labelled with p63 and the basal cell cytokeratin CK5 confirmed the presence of clusters of aberrant basal cells that protrude into the lumen (Figure 4D). *Brca2;Trp53* homozygous mutant HG PIN lesions were then double labelled with CK5 and CK8, a marker of differentiated luminal cells. Areas of neoplasia showed an increase in CK8-expressing luminal cells that were often adjacent to a population of expanded CK5-expressing basal cells (Figure 4D). These regions occasionally had cells that were labelled with both CK8 and CK5, similar to human TA cells that co-express basal and luminal markers, which are not seen in control mouse prostates (Figure 4D) [36].

### *Brca2;Trp53* PIN lesions proliferate post-castration

**Figure 5:**
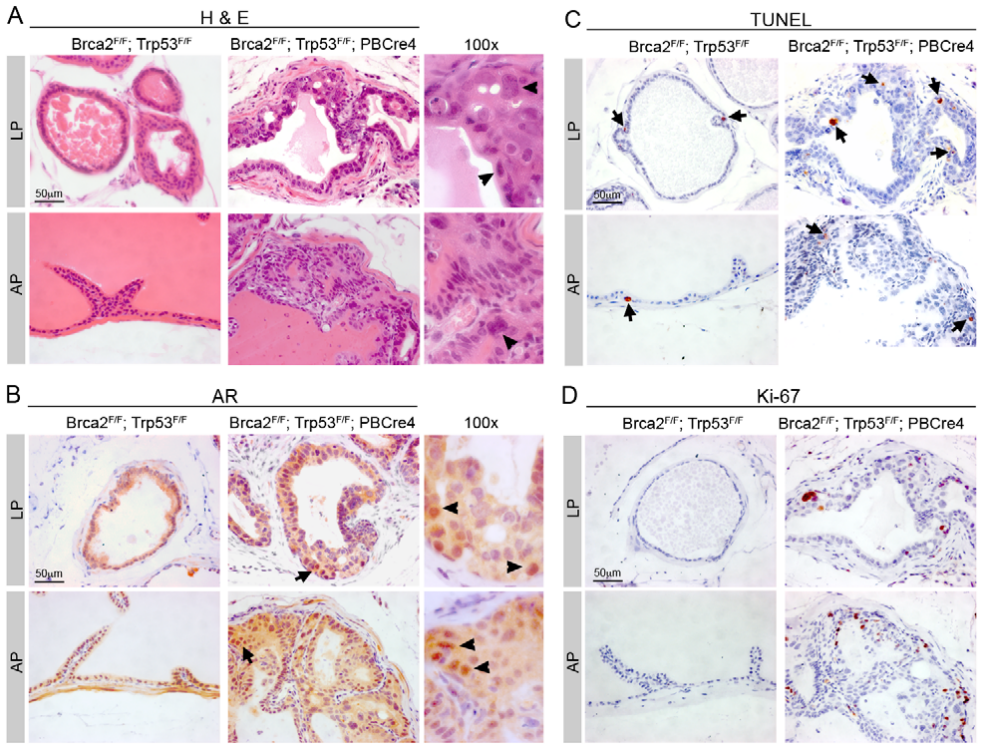
*Brca2;Trp53* PIN lesions proliferate post-castration. Control (*Brca2^F/F^;Trp53^F/F^*) and mutant *Brca2^F/F^;Trp53^F/F^;PBCre4* prostates were surgically castrated at 16 months and culled 4 days later. (A) Haematoxylin and eosin stain shows atypical cells are still present in *Brca2^F/F^;Trp53^F/F^;PBCre4* mutant prostates 4 days after castration, but there is a reduction in cells present in the lumen. Right panels show detail of neoplasia, atypical cells are indicated with arrowheads. (B) AR immunohistochemistry shows expression 4 days post-castration is predominantly throughout the cytoplasm of control and *Brca2^F/F^;Trp53^F/F^;PBCre4* prostates. Arrows indicate cells that have higher nuclear AR expression. Right panels show detail of cells with nuclear AR expression, indicated with arrowheads. (C) TUNEL assay analysis demonstrates an increase in apoptotic cells in control and mutant prostates after castration. Arrows indicate some apoptotic cells. (D) Ki-67 immunohistochemistry shows there are proliferating cells in castrated *Brca2^F/F^;Trp53^F/F^;PBCre4* mutants. LP is lateral prostate, AP is anterior prostate.

Androgen ablation is the standard treatment for human prostate cancer. To assess the response of neoplasias formed after deletion of *Brca2* and *Trp53* to androgen ablation, we surgically castrated animals at 16 months when HG PIN lesions have already formed and analysed them 4 days post-castration. Castration of control animals resulted in normal prostate regression, with a reduction in lumen size (Figure 5A). *Brca2^F/F^;Trp53^F/F^;PBCre4* animals still contained focal areas of neoplasia with atypical cells following androgen-depletion (8/8 mutant animals) (Figure 5A). However, we did not observe ducts with filled lumens as seen in areas of PIN in non-castrated mutant animals.

Following castration of control male mice, AR is expressed at low levels predominantly throughout the cytoplasm of luminal and stromal cells (Figure 5B). Castration of *Brca2^F/F^;Trp53^F/F^;PBCre4* animals resulted in AR expression in a diffuse pattern throughout most prostate luminal and stromal cells (Figure 5B). However, AR is expressed at higher levels in the nucleus of a small number of cells in the PIN lesions of mutant animals post-castration (Figure 5B).

A reduction in the level of circulating androgens results in apoptosis of the AR-expressing luminal epithelial cells, normally leading to initial prostate tumour regression. As expected, luminal cells of control castrated animals contained apoptotic cells throughout the prostate post-castration (Figure 5C). Castrated *Brca2;Trp53* homozygous mutants contained cells in all prostatic lobes undergoing programmed cell death, including apoptotic cells in PIN lesions (2.2% TUNEL positive cells vs 1.8% in castrated control, p = 0.49) (Figure 5C).

Interestingly, Ki-67 staining showed an 18 fold increase in proliferating cells in areas of neoplasia in *Brca2;Trp53* mutants that persisted after castration, compared to control castrated animals (5.5% Ki-67 positive cells vs 0.3% in castrated control) (Figure 5D).

## Discussion

Studies on human carriers of deleterious *BRCA2* mutations have implicated this gene in prostate cancer etiology, but its function in this malignancy is unclear. We have undertaken a genetic analysis of *Brca2* function in the adult mouse prostate to define its role in prostate cancer and to create an *in vivo* model of *Brca2*-dependent prostate disease progression. Our study has demonstrated that loss of *Brca2* in the mouse prostate epithelium results in hyperplasia and LG PIN. These lesions have an increase in the number of cells with DNA damage and apoptotic cells, which could be the result of the impairment of DNA repair pathways. This demonstrates not only that *Brca2* can play a role in the initiation of prostate neoplasia but also that other factors are required for prostate tumour progression.

**Figure 6:**
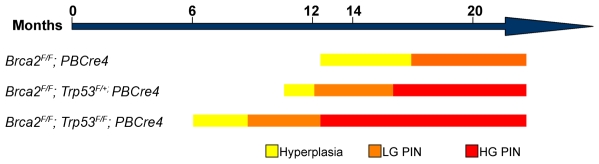
Schematic showing the progression of prostate neoplasia in *Brca2* and *Brca2;Trp53* mutant animals. Note that prostate neoplasia onset is earlier and the severity of PIN increases in *Brca2* mutants as *Trp53* is lost.

Deletion of *Brca2* and *Trp53* in mouse prostate epithelia resulted in a shorter latency and increased frequency of prostate neoplasia compared to deletion of *Brca2* alone (Figure 6). Moreover, the severity of neoplasia increased in *Brca2;Trp53* mutants, with the formation of hyperplasia and LG PIN at initial stages followed by a high incidence of multi-focal, proliferative HG PIN lesions with progressive cellular atypia (Figure 6). HG PIN lesions that form after deletion of *Brca2* and *Trp53* contained many cells with DNA damage, indicating increased genomic instability [17]. Multi-focal lesions are a common feature of human prostate cancer and may be due to defects in the DNA damage response [37]. The formation of HG PIN lesions in *Brca2;Trp53* mutant prostates may reflect the loss of key regulatory p53-dependent functions in response to DNA damage controlling cell-cycle checkpoints, apoptosis and senescence [38]. This demonstrates a co-operative tumour suppressor function of *Brca2* and *Trp53* in the prostate similar to the mammary gland [24]–[26].

A recent study investigating whether *TP53* and *BRCA2* are frequently mutated together in human prostate cancer found that TP53 overexpression could not distinguish *BRCA2* carriers with prostate cancer from a control group of prostate cancer cases [39]. However, this study was limited by the small number of *BRCA2* cases and inability to detect *TP53* mutations that do not stabilize the protein, which are frequently detected in tumours with impaired homologous recombination [40].

Deletion of *Brca2* in other tissues frequently leads to an increase in apoptosis, which is partially or fully rescued upon loss of *Trp53* [31], [32], [41]. However, we see more apoptotic cells in *Brca2;Trp53* mutant HG PIN lesions than in *Brca2* mutant LG PIN lesions. The increase in apoptosis in HG lesions could be due to the rapid accumulation of additional mutations by proliferating cells, which causes catastrophic amounts of DNA damage. This suggests that while some *Brca2*-null cells may be rescued from apoptosis after loss of *Trp53*, other cells in *Brca2;Trp53* deficient HG neoplastic lesions undergo p53-independent cell death. Several different DNA damage-induced p53-independent mechanisms of apoptosis have been reported in different cell types [42].

AR expression is increased in the nucleus of cells in *Brca2;Trp53* HG PIN lesions suggesting they are androgen sensitive. Consistent with this, castration of *Brca2^F/F^;Trp53^F/F^;PBCre4* animals led to regression of PIN and a reduction of cells within the lumen. This suggests that *BRCA2*-driven prostate cancer would initially respond to conventional androgen ablation. However, atypical cells persist in *Brca2;Trp53* HG PIN lesions that continue to proliferate, indicating these lesions may be able to re-grow and become castration-resistant. Interestingly, some cells in *Brca2;Trp53* mutant PIN lesions expressed AR at higher levels in the nucleus after castration, indicative of active AR signalling. Castration-resistant human prostate cancer growth commonly remains AR-dependent and is thought to occur through several mechanisms including *AR* amplification, AR mutation, changes in AR co-regulators and growth factor activation [43]. The presence of nuclear AR in castrated *Brca2;Trp53* mutant prostates may indicate the regulation of proliferation by this factor after androgen depletion.

We often observed an increase in p63 positive basal cells, the presence of TA-like cells and an adjacent expansion of luminal cells in *Brca2;Trp53* mutant HG PIN lesions. This suggests increased proliferation of the basal cell population, with maintenance of differentiation into luminal cells. Similarly, deletion of the tumour suppressor *Pten* with the *PBCre4* transgene results in tumour formation with an increase in basal cells that contain a progenitor cell sub-population, the presence of TA-like cells and luminal cell differentiation [44]. The increase in basal progenitor cell population could represent an expansion of cancer-initiating cells, indicating that HG PIN lesions in *Brca2;Trp53* mutant prostates may originate from these cells. Several other murine models of prostate cancer that utilize the *PBCre4* transgene display increased and aberrant p63 expression during early stages of cancer progression [29], [30]. This transgene is expressed in both the basal and luminal cells of the mouse prostate [44]. In contrast, deletion of *Pten* using a *PSA*-driven *Cre* only expressed in luminal cells results in cancer without an expansion in p63 cells [45]. However, this model has slower kinetics than the *Pten*; *PBCre4* model and tumours are initiated from cells in the luminal epithelial compartment. Frequently during human prostate tumour progression there is an increase in TA/progenitor cells, which has led to the proposal that these cells could be tumour-initiating cells [36], [44], [46]. Taken together, these data suggest that an increase in the basal progenitor cells could be a common early event in prostate neoplasia but may be dependent on the origin of the cancer-initiating cell.

Although we frequently observed HG PIN lesions in *Brca2;Trp53* mutant prostates no invasive carcinoma formed. In contrast, deletion of *Brca2* and *Trp53* in the mouse mammary gland results in invasive carcinoma at 6 months, and consistent with this, human carriers of *BRCA2* mutations have a high-risk of breast cancer. The lack of prostate carcinoma in our mutants may reflect the relatively low penetrance of prostate cancer in human *BRCA2* mutation carriers and suggests there is only a subset of *BRCA2* carriers that develop aggressive forms of the disease [13]–[16]. This subset may be dependent on additional genetic modifiers or environmental factors that influence the risk of individuals carrying a *BRCA2* mutation forming prostate tumours [47]. It is possible that human carriers of deleterious *BRCA2* mutations frequently form HG PIN lesions similar to our *Brca2;Trp53* model but that never progress to carcinoma and therefore go undetected. Ongoing work into genetic modifiers of *BRCA2* may identify which subgroups of patients with *BRCA2* mutations are more at risk of developing aggressive forms of the disease.

Variations in genetic background can have a modifying effect on prostate tumour development in mice with tumour suppressor deletions [48]. Due to the complex nature of mouse breeding we were not able to investigate the effects of genetic background on the prostate lesions observed in *Brca2;Trp53* mutant mice in this study. Although a change in genetic background may alter the frequency of tumour phenotype, we only observed PIN in mutant animals suggesting these lesions are an effect of *Brca2* and *Trp53* loss.

This murine study has demonstrated that deletion of the tumour suppressor *Brca2* results in LG PIN, with the additional loss of a second tumour suppressor *Trp53* leading to HG PIN. Other mouse models of tumour suppressor gene loss result in varying degrees of prostate tumour progression. Mice with prostate-specific homozygous *Pten* deletion progress to invasive carcinoma and metastasis [28]. The further loss of *Trp53* in this model results in a shorter latency to invasive carcinoma [49]. Deletion of *Nkx3.1*, a gene involved in prostate epithelial cell differentiation, leads to mice that develop epithelial hyperplasia and dysplastic lesions that resemble human PIN, but do not progress to invasive carcinoma [50], [51].

Our pre-invasive model could be used in the future to test the response to potential therapeutic agents and combination therapies. For example, a recent synthetic lethal approach using PARP inhibitors has been used successfully to specifically induce cytotoxicity in HR-deficient cells [52]. Promising phase I clinical data in *BRCA2* carriers with a PARP inhibitor has shown antitumour activity, including resolution of bone metastases in one patient with prostate cancer [53]. These *Brca2* mutant mice may provide a useful model to examine cellular responses, such as apoptosis, to combinations of therapies for optimisation of treatment. In addition, this study demonstrates that *Brca2* acts as a tumour suppressor and can interact genetically with *Trp53* deficiency in the prostate preventing DNA damage accumulation and neoplasia progression.

## Materials and Methods

### Ethics statement

Animals were handled in strict accordance with UK Home Office regulations.

### Generation of prostate-specific *Brca2* and *Trp53* deletion mice

*Brca2^F/F^* (targeting exon 11) mice and *Trp53^F/F^* (targeting exons 2–10) mice [25] and *ARR2PBCre* transgenic mice, *PBCre4* [27], have been previously described. The animals were bred on a mixed genetic background.

### Mouse prostate histology and statistical analysis

Histological phenotype of samples was assessed on haematoxylin and eosin stained sections. Serial sections were then stained for immunohistochemical analysis. Histological assessment was based on published guidelines and assisted by a pathologist [54], [55]. PIN lesions noted as LG were equivalent to PIN I-II and those noted as HG were equivalent to PIN III-IV in Park et al [54]. The two-sample Z-test was performed to test if there is a significant difference between groups of animals.

### Quantification of proliferation and cell death

Ki-67 or TUNEL staining was performed by immunohistochemistry on sections and cells stained with nuclear brown DAB chromogen were counted as positive. Cells from at least 4 high power fields were counted per animal, which totalled more than 900 cells per animal. Five animals of each genotype were analysed. Randomly selected fields were counted for control analysis and sections corresponding to histologically identified areas of hyperplasia and PIN were counted for mutant animals. All values are significant with p<0.05 using Student t-test unless otherwise stated.

### Immunohistochemistry analysis

Antibody stains were done on paraffin sections as previously described [56]. The following antibodies were used: Ki-67 (TEC-3, Dako, 1∶200 with amplification), AR (PG-21, Upstate, 1∶250 with amplification), p63 (4A4, Santa Cruz Biotechnology, 1∶200 with amplification, 1∶50), γH2AX (JBW301, Upstate, 1∶800 with amplification), CK5 (PRB-160P, Covance, 1∶50), CK8 (MMS-162P, Covance, 1∶200). The ABC elite vector kit was used for amplification using biotinlyated secondary antibodies (Vector Laboratories) and the DAB substrate (Dako). Secondary fluorescent antibodies were obtained from Molecular Probes and were used at a 1∶1000 dilution. TUNEL analysis was carried out using the ApopTag apoptosis detection kit (Chemicon).

## Supporting Information

Figure S1 *Brca2* and *Trp53* are deleted in mutant adult prostates. PCR analysis of *Cre*, *Brca2*, and *Trp53* on dissected anterior prostate (AP), lateral prostate (LP), dorsal prostate (DP), and ventral prostate (VP) tissue from a *Brca2^F/F^;Trp53^F/F^* (control) *and Brca2^F/F^;Trp53^F/F^;PBCre4* (mutant) animal. Wild type (WT) and water (H_2_0) are controls.
(0.07 MB DOC)
