# Supplementary figures and images for: Brca2 and Trp53 Deficiency Cooperate in the Progression of Mouse Prostate Tumourigenesis
Source: PLoS Genet. 2010 Jun 24;6(6):e1000995. doi: 10.1371/journal.pgen.1000995 (PMC2891704; doi:10.1371/journal.pgen.1000995)

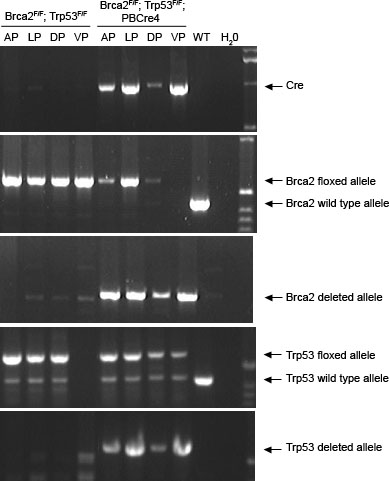

Supplement: Figure S1 — Brca2 and Trp53 are deleted in mutant adult prostates. PCR analysis of Cre, Brca2, and Trp53 on dissected anterior prostate (AP), lateral prostate (LP), dorsal prostate (DP), and ventral prostate (VP) tissue from a Brca2F/F;Trp53F/F (control) and Brca2F/F;Trp53F/F;PBCre4 (mutant) animal. Wild type (WT) and water (H20) are controls. (0.07 MB DOC) [file pgen.1000995.s001.doc]
